# Supplementary material for: Patterns of Intron Gain and Loss in Fungi
Source: PLoS Biol. 2004 Nov 30;2(12):e422. doi: 10.1371/journal.pbio.0020422 (PMC532390; doi:10.1371/journal.pbio.0020422)
Supplement: Table S1 — Also available at http://genes.mit.edu/NielsenEtAl/. (4.3 MB ZIP). [file pbio.0020422.st001.zip › NielsenEtAl/html/96.html]

AN4975.1.NCU04771.1.MG08877.1.FG08428.1


```
 CLUSTAL W (1.82) Multiple Sequence Alignments - Introns Inserted


Sequence 1: NCU04771.1	502 aa
Sequence 2: MG08877.1	475 aa
Sequence 3: FG08428.1	473 aa
Sequence 4: AN4975.1	441 aa
Alignment Length: 522 aa
Number Identitical Residues: 134 aa
Alignment Score (without introns) 7501


MG08877.1 	MSSKSSYSN-------QRSN-------TPSSILIVGSGVFGL~GTAYALARRDAYSNTTI
NCU04771.1	MATPSAPSSSLPPPKPQQSSSASSPLSPPSSILIIGSGVFGL~TTAYALSRRHDFVNTTI
FG08428.1 	MSG------------------------APSSILIVGSGVFGL~GTAWALTKRPQYSNTKI
AN4975.1  	MSL-------------------------PDNILIVGGGVFGL1STALSLSKR--HPNSKV
          	*:                          *..***:*.*****  ** :*::*  . *:.:

MG08877.1 	TVLDRS--VDEALFPSRDASSIDTSRIIRADYADPAYAALAAEAQHEWRKQGD-DDLGGQ
NCU04771.1	TVVDRADPSNPDVFPAPDAASVDTSRIIRADYPDAAYAALAAEAQLQWRQQTHPDDLGSE
FG08428.1 	TIVEEC----AGQFPPEDGASVDSSRIVRADYSDPHYAALAAEAQKEWRKQGD-DEVGGQ
AN4975.1  	TLLEAS-----PTIPNPHGSSVDSSRIIRADYSNPTYAKLASEAVDLWR----TTEWGHE
          	*::: .       :*  ..:*:*:***:****.:. ** **:**   **    . : * :

MG08877.1 	GRYSESGLVLVVDN--------VPSG----AAAKKDGAYYCKQSWENVNAMAEKDPSLQG
NCU04771.1	GRYSESGLLVVADGPAPPPGTPVAPGTSVIDKSKLTGMDYARFSWANVLSLASSDPELAA
FG08428.1 	GRYSESGFVLCASE--------TPKD----FKMKKSGMDYTKESAKNVELIAAQTGLPMD
AN4975.1  	GRYTQNGLLLVYPD------------------GNSTAKDYTTKSYHNVKQLGENAQFLPT
          	***::.*:::                       :  .  *   *  **  :. .      

MG08877.1 	KLKLLPNIEAIRDAVGTGHVNSGVWGYQNLNSGWADADRSMQWLLRKVKETGRVKFVSGT
NCU04771.1	RIKECPNTDAIKAALGTGGS-SGSWGYINGLSGWANAGASMAWLYKRVRAEGRINFVAGE
FG08428.1 	KIQKLESTKALQEFLGTDGY-PGDWGYLNGNSGWADAGEGMKWLYKQASATGRIEFVNGK
AN4975.1  	KADVLKAAPAYGETLQVAGG------YVNWSSGWADAEAGVRFAKQKLDKEGKVRFVHGE
          	: .      *    : .         * *  ****:*  .: :  ::    *::.** * 

MG08877.1 	AASLNR---DGKKITGVKLSDGR--QLSADLVVLATGAWTGGLVDLAGRAAATAQVLAYL
NCU04771.1	VTNLEV---SGNTVTGARLSDGR--VLSADLVMVSAGAWTGRLVDLTGQAIATGQVLGYI
FG08428.1 	VTELVT---EGDRVTGAKLSDSK--VLKAEVVMVAAGAWSGSLVDLRGRTEATGHAVTYM
AN4975.1  	VSRLLFSPIDNRTVTGVVLKDFKNTTMTADLVILATGAWTAGLVDLRGRAVSTGQAIAYM
          	.: *  :. ..  :**. *.* :.: :.*::*::::***:. **** *:: :*.:.: *:

MG08877.1 	DITDEEQAALAEMPVYLNFGSGLFIIPPANNVLKVARHEYGYLNPTTNATPLRSG-TSSD
NCU04771.1	DLTPEEEAQLAHMPVILNLSTGLFVIPPRNGVLKVARHAYGYLNPTTLSVPPLAS-FVTT
FG08428.1 	DITSEEQRRLDNFPVVLNLSTGLFLIPPRNNVLKVARHTFGYINPVKITNALPPSPNDKR
AN4975.1  	RISDEEQRRLEHMPTILNFATGIFIIPPRNNILKIARHAYGYHNPKAVPVPGATP---TG
          	 :: **:  * .:*. **:.:*:*:*** *.:**:*** :** **   . .  .    . 

MG08877.1 	APYTVSLPPGDVAEAFQDIPQESKEALRSALRDMVPIPAIHDRPFTSTRVCWYTDT~KSG
NCU04771.1	PAAAVSLPLTTLTDPTLQIPTEGADDLRRALHEMVPLPSLRDRPFSKTRICWYSDT~PTA
FG08428.1 	EAFIASQPYTSRNSSENPLSVEADKDLRRALKDLCPVRGLETRPWKEARICWYSDT~RDG
AN4975.1  	ETMHVSLP-----EKGVPIPLEGETAFRTALKQLLPS--FADRPFLNTRICWYTDT2PRG
          	 .  .* *     .    :. *.   :* **::: *   :  **: .:*:***:**   .

MG08877.1 	DFIIDYHPEWEGLFLATGGSGHGFK0FLPVLGERIADCIAYDRPSEFAEKWAFKSLPDDV
NCU04771.1	DFIVDYHPKYKGLFVATGDSGHAFK~FLPVIGEKIADVIAGQCPPEFVGKWNWRYPASDA
FG08428.1 	EWLIDYHPGWKGLFVATGDSGHGYK~FLPNLGEKIADVLQGQG-GELGEKWRWREMKNDG
AN4975.1  	DFIITHHPSHPHLVLATGGSGHAYK~FFPLIGEKVVDALEGKLAPELRALWDWPEAVDEA
          	:::: :**    *.:***.***.:* *:* :**::.* :  . . *:   * :    .: 

MG08877.1 	R-------WDQVCTEDGSRAGQAGRYFKKELAKSTKTSKL-----
NCU04771.1	A-------IKTVITEDGSRGGVPGLILSTELERTSAVLN------
FG08428.1 	VGRETNGVYNGLMTQDGSRGGRP-LVLCDELAKGRAPVGEAKAKL
AN4975.1  	H---------FDGTEDGSRSGDKRLLLLDELAKGQKAKRESAL--
          	             *:****.*     :  ** :       :
```
